# Is Pulmonary Consumption Contagious?

**Published:** 1887-12

**Authors:** A. Brochin


### IS PULMONARY CONSUMPTION CONTAGIOUS?

BY A. BRCCHIN, M. D.

Translated from *Te Journal de Med. de Paris*, F. R. CAMPBELL, A.M., M.D.

No question is of greater importance to the practitioner and sanitarian than that of the contagiousness of pulmonary consumption. I am surprised that the subject is not more frequently discussed in our scientific societies, and that the report of our eminent confrere, M. Vallin, is not taken as the basis of further researches.

While the profession is sti l divided on this question of the contagiousness of tuberculosis, the number of "contagionists" is daily increasing. If each physician would search through the records of his practice there would be discovered such a number of cases pointing to the propagation of consumption by contagion that the most skeptical would be convinced.

We do not mean to say that consumption is as contagious as small-pox, scarlatina, typhoid fever, or even erysipelas. If it were, we would all die of tuberculosis. But we cannot conscientiously deny that consumption is often propagated from person to person where the surrounding and physical conditions of those exposed are suitable. It is, therefore, the duty of physicians to take certain precautions to arrest the ravages of this dread disease

I will now submit to you some facts tending to demonstrate that pulmonary tuberculosis is contagious. In October, 1885, I was called to attend Madame G., a school teacher, who for sometime had complained of a cough and a feeling of fatigue. She was a lady of 25 years of age, married three years, having had two children. Since her last confinement she had had a cough, and lost weight and strength. A physical examination of the chest revealed undoubted signs of tuberculosis in its first stages. Her grandmother and a sister had both been affected with lung disease. Her duties as teacher in a large school exhausted her very much. She lived with her husband in the basement of the school building, in rooms badly lighted and ventilated. The disease increased so rapidly that in January, 1886, her husband informed me that she passed whole nights in coughing and spitting, and her perspiration was so excessive that it was necessary to change her clothing several times every night.

She died in March, and her husband, who was absolutely without any tendency to consumption, as far as family history and physical conformation was concerned, became tuberculous and at this writing, March, 1887, has an enormous cavity in his left lung and tubercular granulations in the larynx.

This fact appears to me to be demonstrated, that a healthy man, without any constitutional predisposition to phthisis, if exposed for a long period to the exhalations of a tubercular patient, will acquire the disease himself if the hygienic surroundings are unfavorable.

CASE II.—Madame Y., aged 31 years; father and mother died of consumption, became tuberculosis herself, and died after an illness lasting four years. Her husband, aged 33, was a strong, healthy man, without any history of tuberculos in his family, occupied the same room with his wife up to three months before her death. Even before she died he was affected with a cough, lost his appetite, became emaciated, and had night sweats. On auscultation I discovered a consolidation at the apex of the left lung. How could the disease have been acquired except by contagion?

CASE III.—Madame H.; aged 25 years, married three years, two children, exhibited signs of tuberculosis in November, 1884. After careful and prolonged treatment, her condition improved and she renewed her household duties, but continued to cough. Her husband, aged 30 years, hitherto in the best of health and without any hereditary tendency to lung disease, was attacked with what was apparently acute bronchitis. But alarming symptoms of consumption soon developed, and in spite of energetic treatment he died of phthisis in July, 1886. His wife is still living, but has a cavity in her left lung. Her children are in good health.

CASE IV.—This case relates to the history of one entire family. The father, aged 35, printer by trade, was obliged to work many hours a day to support his wife and six children. Of these children, two died of tubercular meningitis when two years of age. The third year the father became ill, and died of consumption in six months. The eldest son, who assisted his mother in the house, died of acute tuberculosis. This was the fourth death from tuberculosis in three years in this family. You will say that heredity might explain all, but how will you account for the following:

The mother, an extremely robust and energetic woman, with no hereditary tendency to consumption, continued to do her utmost for her husband and children. She soon became phthisical, and in a short time died. One of the remaining three children, a girl 16 years of age, is already pale, emaciated, and seems to be tuberculous.

In this history, we can explain the disease of the father, who was the son of a consumptive, and of the children by heredity. But what other cause than contagion can be invoked to explain the affection of the mother? Will you say that overwork and grief was the cause? These may be contributing causes, but are not sufficient to explain the appearance of tuberculosis in a robust woman, hitherto in the best health, and without a family history of the disease.

It is a generally received opinion among contagionists that death comes on more rapidly in cases where the disease is acquired by contagion than where it is primarily developed. I do not know whether these rules stand the test of further investigation, but in the cases just reported this seems to be true. But I have observed a number of cases in which children, attending parents afflicted with tuberculosis, would appear to have signs of the disease, which were in time relieved.

I will cite, for example, the case of a young lady, 20 years of age, who devoted her whole time to the care of her father, who died of consumption. She remained in the rooms which had been occupied by her father, and became ill with what appeared to me and other physicians to be consumption. But she has since regained her health, and earns her living by her own hands. Of course, we may have been mistaken in our diagnosis, for the cure of consumption in the condition of poverty in which this girl lives is very rare indeed,

I could give many more examples of the contagiousness of consumption which I have observed in my practice during the last fifteen years. But it is a subject so extensive that prolonged study and deep research are necessary to clear up all the factors in these cases. I have only desired to call your attention to some facts pointing to the contagiousness of phthisis, and I would be very happy if other members of the profession would publish their observations on this subject.

If it can be demonstrated beyond question that tuberculosis is contagious, it becomes our duty to provide separate wards for consumptives in hospitals; to insist on the isolation of our patients in private practice, and to employ disinfection and antiseptics in the treatment of these cases, matters which have been hitherto entirely neglected in this country, although carried out in Germany and England.—*Buffalo Medical and Surgical Journal.*

